# Scanning WAXS microscopy of regenerated cellulose fibers at mesoscopic resolution

**DOI:** 10.1107/S205225252400383X

**Published:** 2024-06-11

**Authors:** Sara Johansson, Francesco Scattarella, Sebastian Kalbfleisch, Ulf Johansson, Christopher Ward, Crispin Hetherington, Herbert Sixta, Stephen Hall, Cinzia Giannini, Ulf Olsson

**Affiliations:** a Division of Solid Mechanics Lund University Box 118 LundSE-22100 Sweden; b Institute of Crystallography National Research Council Via Amendola 122/O Bari70126 Italy; c MAX IV Laboratory Lund University PO Box 118 LundSE-22100 Sweden; d Physical Chemistry Lund University Box 124 LundSE-22100 Sweden; e National Center for High Resolution Electron Microscopy, Centre for Analysis and Synthesis Lund University Box 124 LundSE-22100 Sweden; f Department of Bioproducts and Biosystems Aalto University PO Box 16300 Helsinki00076 Finland; SPring-8, Japan

**Keywords:** nanofocus X-ray probe, scanning WAXS, regenerated cellulose fibers, materials science, crystallization, crystal growth, properties of solids

## Abstract

In this work, regenerated cellulose textile fibers, Ioncell-F, dry-wet spun with different draw ratios, have been investigated by scanning wide-angle X-ray scattering (WAXS) using a mesoscopic X-ray beam. The fibers were found to be homogeneous on the 500 nm length scale. Analysis of the azimuthal angular dependence of a crystalline Bragg spot intensity revealed a radial dependence of the degree of orientation of crystallites that was found to increase with the distance from the center of the fiber. We attribute this to radial velocity gradients during the extrusion of the spin dope and the early stage of drawing. On the other hand, the fiber crystallinity was found to be essentially homogeneous over the fiber cross section.

## Abstract 2

Scanning WAXS microscopy of a regenerated cellulose textile fiber reveals a radial gradient in the degree of orientation of the crystallite.

## 1. Introduction

Wood-based cellulose is an important and renewable raw material for various materials (Klemm *et al.*, 2005 ▸; Wang *et al.*, 2016 ▸; Tu *et al.*, 2021 ▸). One example is textile fibers (Woodings, 2001 ▸; Sixta *et al.*, 2015 ▸), where man-made regenerated cellulose fibers represent an alternative to natural fibers such as cotton and polyesters. Cellulose is a crystalline polymer with a high melting point that cannot be reached without chemical decomposition. Therefore, shaping cellulose into a particular material generally requires an initial dissolution step. This is also a considerable challenge due to the highly stable crystalline structure combined with high molecular weight. These two factors together make cellulose fascinatingly insoluble in all simple molecular solvents, from polar to non-polar. However, strong alkaline aqueous solutions titrate the glucose –OH groups (Marsh & Wood, 1942 ▸; Bialik *et al.*, 2016 ▸) if one can suppress the crystallization of cellulose salts (Gubitosi *et al.*, 2017 ▸; Martin-Bertelsen *et al.*, 2020 ▸). The century-old viscose process involves derivatization of cellulose to a xanthogenate as a means to avoid crystallization and increase the effective solubility. Certain ionic liquids (Swatloski *et al.*, 2002 ▸; Idström *et al.*, 2017 ▸) and amine oxides (Rosenau *et al.*, 2001 ▸) are also known to solubilize cellulose and have been applied for processing textile fibers. Here, a relevant example is the well known Lyocell fiber, produced from amine oxide *N*-methyl­morpholine *N*-oxide (NMMO) monohydrate solutions (Rosenau *et al.*, 2001 ▸; Röder *et al.*, 2009 ▸).

The relationship between the internal material structure, typically on the nanometre or colloidal length scale, and the mechanical properties of the material, is often a key question in materials science and process engineering. Exploring and understanding such relationships require detailed structural characterization on the nanometre length scale. Such information is typically obtained by employing X-rays, electrons or neutrons. We have recently performed a detailed study of the colloidal structure of regenerated cellulose textile fibers (Ioncell-F) using a combination of small- and wide-angle X-ray scattering (SAXS/WAXS) (Gubitosi *et al.*, 2021 ▸). These fibers were produced by dry jet wet spinning with different draw ratios from a concentrated cellulose solution in the ionic liquid [DBNH][OAc]. The results suggested an internal structure consisting of disk-like crystalline domains embedded in an amorphous matrix. The WAXS data confirmed a significant orientational order of the crystalline domains, with the cellulose chains parallel to the fiber direction (Gubitosi *et al.*, 2021 ▸). The experiments were also able to characterize the anisotropy of the amorphous domains. Furthermore, by integrating the individual scattering contributions from crystalline and amorphous domains, an accurate estimate of the fiber crystallinity could be obtained (Gentile *et al.*, 2022 ▸). The experiments were performed by means of an in-house laboratory source pinhole SAXS/WAXS instrument with the X-ray beam collimated to a cross section size of *ca* 500 µm (*i.e.* many times larger than a typical fiber diameter), and the experiments were performed on bundles of parallel fibers. Thus, the results obtained by Gubitosi *et al.* (2021 ▸) and Gentile *et al.* (2022 ▸) were averages over large fiber volumes including the whole fiber cross section.

Extruded fibers typically have cylindrical symmetry. Being homogeneous in the fiber direction one may expect, for example, due to processing conditions, a radial gradient in structural properties perpendicular to the fiber axis. This can be explored by scanning experiments on single fibers using a small (micro- or nano-) X-ray beam (Riekel & Davies, 2005 ▸; Riekel *et al.*, 2010 ▸). The pioneering works of Riekel and coworkers (Davies, 2003 ▸, Davies *et al.*, 2006 ▸; Roth *et al.*, 2003 ▸) indeed demonstrated the possibility to measure structural variations on the sub-micrometre length scale in addition to the local strains as effects of fiber tensile deformations.

In the present paper, we revisit the Ioncell-F fibers and focus on possible structural inhomogeneities within single fibers due to processing details. Here, we have applied scanning WAXS measurements of three different fibers, produced with different draw ratios, with a final spatial resolution of 500 nm. This resolution is significantly larger than the smallest fiber radius, allowing us to evaluate possible radial gradients in structural parameters. In the fiber-spinning process, the dissolved and drawn cellulose is regenerated into fibers by precipitation (coagulation) in a spinning bath consisting of water and accumulated solvent. After contact with the anti-solvent in the spinning bath, there is a radial gradient in the solvent composition, and precipitation is expected to begin at the interface and propagate towards the center. For this reason and because of the velocity gradients during extrusion, it is possible that the fiber structure may vary in the radial direction. Finally, this study also offers an interesting test of the challenges involved when investigating radiation-sensitive organic materials with a highly brilliant X-ray beam at a fourth-generation synchrotron.

## 2. Materials and methods

### 2.1. Materials

The fibers were produced by dry jet wet spinning, as described in detail by Asaadi *et al.* (2018 ▸). In short, a spin dope composed of 13 wt% solution of cellulose in the ionic liquid [DBNH]OAc was extruded through a 36 hole spinneret with a capillary diameter *D* = 100 mm and length *L* = 20 mm (*L*/*D* = 0.2), and then drawn with different draw ratios. Three different draw ratios of 1, 7 and 15 referred to below as DR1, DR7 and DR15, respectively, were selected and investigated here. The corresponding fiber diameters were 36, 15 and 10 µm, respectively.

### 2.2. Data acquisition

The fibers were investigated with scanning WAXS microscopy at the NanoMAX beamline, MAX IV Laboratory, Lund, Sweden (Johansson *et al.*, 2021 ▸; Carbone *et al.*, 2022 ▸). The beamline is a hard X-ray scanning nanoprobe beamline with multiple detection methods. The current experiment was carried out at 12 keV photon energy with the beam focused to 100 nm and with a beam intensity of approximately 8 × 10^8^ photons s^−1^. The beamline can provide much higher intensity, but to avoid immediate radiation damage to the fibers, the intensity was attenuated. The highest acceptable intensity was determined by repeatedly scanning the same area of a fiber and then visually observing at what level the diffraction pattern did not change with repeated scans.

**Figure 1:**
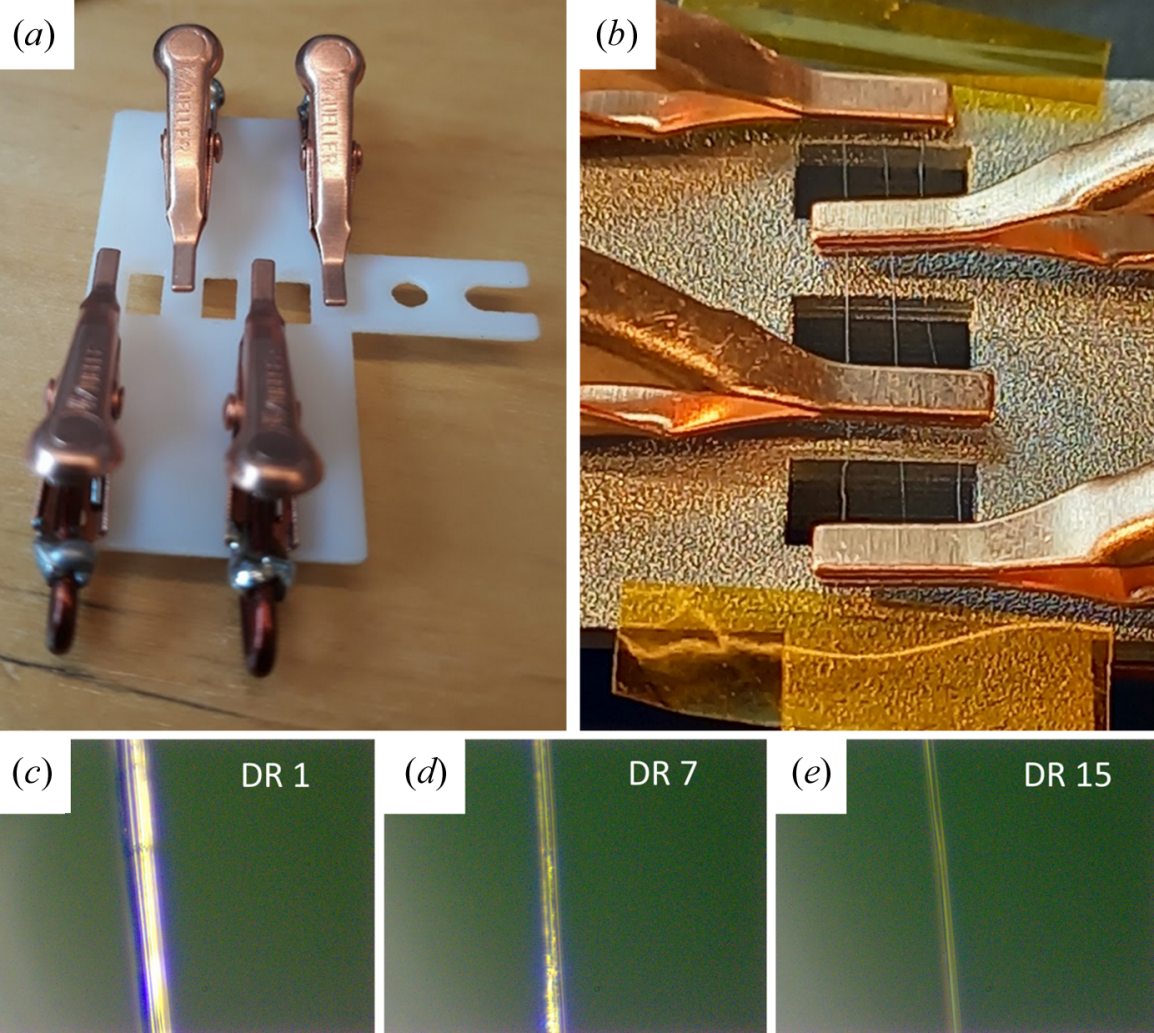
Experimental setup. (*a*) Photograph of the sample holder with clamps used to fix the fibers. (*b*) Close-up under the microscope with the thin fibers visible. (*c*)–(*e*) Photographs of each fiber during the WAXS measurements. The diameters of the fibers are (*c*) 36 µm, (*d*) 15 µm and (*e*) 10 µm.

Figs. 1 ▸(*a*) and 1 ▸(*b*) show photographs of the fibers mounted in the sample holder, and Figs. 1 ▸(*c*)–1 ▸(*e*) are photographs of each fiber, acquired through the in-line optical microscope during the WAXS measurements. The fibers were mounted vertically, and each fiber was scanned through the beam in the horizontal direction as the fast axis and slowly stepping in the vertical direction for each horizontal line. The horizontal scanning was done at constant speed, and the WAXS detector was triggered at intervals to acquire diffraction patterns. The resulting dataset consists of an *XY*-array of diffraction images. All datasets were acquired using horizontal scans with a 100 × 100 nm beam, a continuous scan rate of 100 nm/0.3 s and frames recorded with an acquisition time of 0.3 s. Hence, these resulted in an effective frame size of 100 × 100 nm. The horizontal scan ranges were 60 µm for the DR1 fiber and 40 µm for the DR7 and DR15 fibers, and a total of 150 lines were scanned for all fibers, giving a 15 µm vertical scan range. The total measurement time for the three fibers was approximately 20 h.

The 2D detector was placed directly downstream of the sample, centered in the beam. A beam stop was placed close to the detector to block the direct intense photon beam. A photon flux monitor (ion chamber) was placed downstream of the nano-focusing optics, before the sample. This signal, *I*_0_, was used in the normalization procedure of the raw data, accounting for possible variation in the primary beam intensity. A silicon powder sample was measured in conjunction with the fiber measurements to accurately determine the sample-to-detector distance and calibrate the *q* scale. Further details about the experimental instrumentation are given in the literature (Johansson *et al.*, 2021 ▸; Carbone *et al.*, 2022 ▸).

### 2.3. Postprocessing and data analysis

Data analysis was mainly performed using MATLAB R2020a, but also by means of a newly updated version of the scientific MATLAB based package *SUNBIM* [supramolecular and submolecular nano- and biomaterials X-ray imaging (Siliqi *et al.*, 2016 ▸)], not yet made public. We exploited *SUNBIM* routines to locate the beam center and calibrate the data using the ‘Calibration’ section. As a first inspection of the datasets, scanning WAXS maps, showing the total number of detector counts in each frame, were constructed in MATLAB. The results (Fig. S1 of the supporting information) showed varying intensities, also outside the fibers (air), and a significant apparent tilt of the fibers, presumably due to small sample movements. To account for a possible variation in beam intensity during scanning, the *I*_0_ signal was used to normalize the intensity of the scattering pattern in each frame. The original datasets were thereafter resampled to account for the tilt (see supporting information). The tilt correction was performed by measuring the apparent tilt angle, whereafter appropriate numbers of pixels were shifted from the beginning to the end of each scan line. The DR15 fiber was not optimally aligned and drifted outside the scanned field of view at the bottom part of the scan. Thus, for this fiber, only *ca* 10 µm in the vertical direction was finally used.

A preliminary signal-to-noise ratio (SNR) analysis of the WAXS patterns was performed using the *S-SAWANA* section of *SUNBIM*, after centering, calibration and folding the original 2D frames into 1D patterns. Each scattering pattern consisted of a low number of detector counts, as a result of the necessary short exposure times. To increase SNR, the datasets were therefore down-sampled in MATLAB by summation of the WAXS patterns in 5 × 5 pixel-sized windows, giving a final spatial resolution of 500 × 500 nm. The results after all of the pre-processing steps are shown in Fig. S2, where it is evident that background intensity variations remained after *I*_0_ normalization. The variations are small (on the order of 1%) and we interpret them as resulting from small variations in the air pressure in the sample area.

The present experiments were performed in air, and the air-scattering results in a significant background that needs to be subtracted. With this large background, small fluctuations become significant. To account for this as well, background subtraction was performed on a line-by-line basis. The WAXS patterns in the first six frames in each scan line were averaged and then subtracted from all WAXS patterns belonging to the same line.

We note in passing that, in principle, one could also use the pure air scattering to calibrate the scattered intensity to absolute scale. Since the pressure (ambient) and the composition of the air are known, the total scattering cross section is known. In the present study, however, such a calibration was not of particular use and was therefore not carried out.

**Figure 2:**
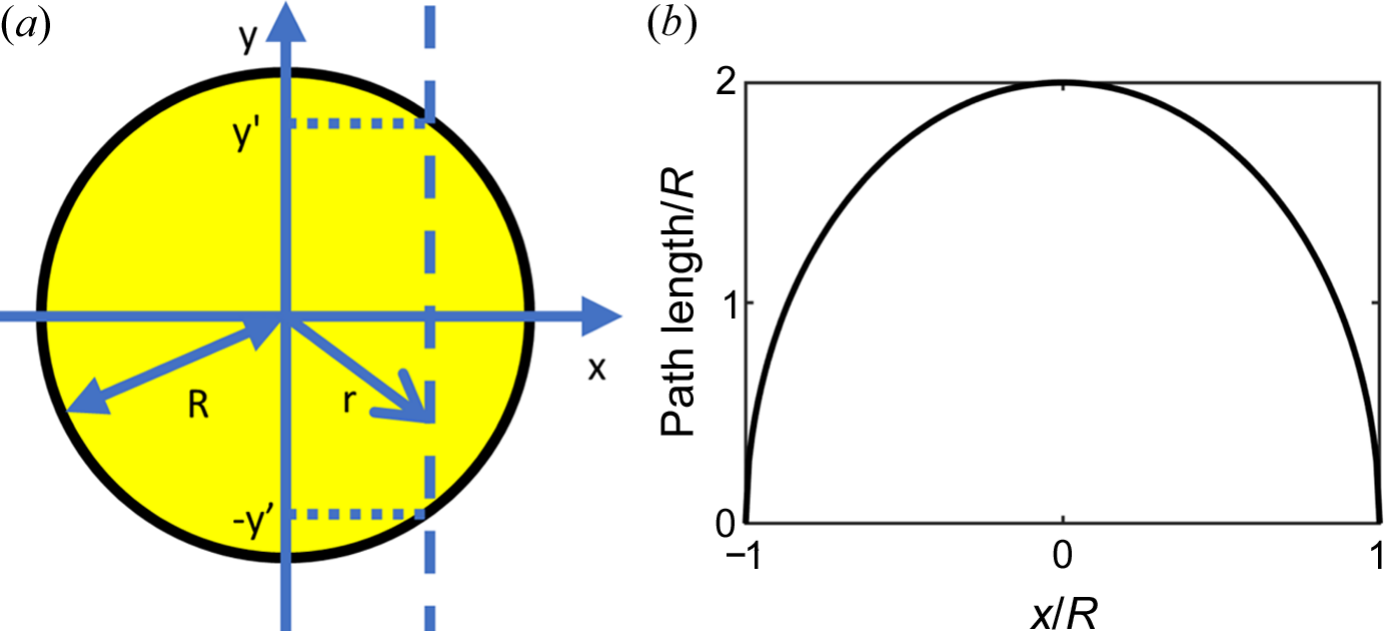
(*a*) Schematic of the circular fiber cross section of radius *R* in the *xy* plane; *r* is the radial coordinate of length 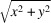 and the broken line represents an example of the X-ray beam position, having the path length 2*y*′. (*b*) Plot of the reduced path length *l*/*R* as function of the reduced *x* coordinate *x*/*R*.

The preprocessed, background-subtracted and normalized scanning WAXS data provide representative and interpretable data for each fiber. However, the intensity varies across the fibers because of the variation in path length through the circular cross section. A path-length normalization processing step, which consisted of normalizing the scattered intensity according to a modeled path length, was therefore performed on the scanning datasets. The purpose was to facilitate an analysis of structural differences in the radial direction. Fig. 2 ▸(*a*) shows a schematic of the circular fiber cross section in the *xy* plane. The primary X-ray beam wavevector, **k**_0_, is in the *y* direction and the fibers were scanned with lines in the *x* direction. The path length [Fig. 2 ▸(*b*)] varies with the *x* coordinate according to
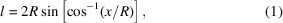
with −*R* ≤ *x* ≤ *R*, *R* being the fiber radius. This path-length variation across the fibers is illustrated in Fig. 2 ▸(*b*). *l* = 2*R* when the beam passes through the center of the fiber and reaches 0 at the edges. A normalization factor was thereafter modeled according to the circular geometry for each pixel center within the fiber.

Finally, azimuthal integration of the WAXS processed data and fit analysis on the resulting intensity plots were performed in MATLAB (see the Results and discussion[Sec sec3] for more details).

## 3. Results and discussion

**Figure 3:**
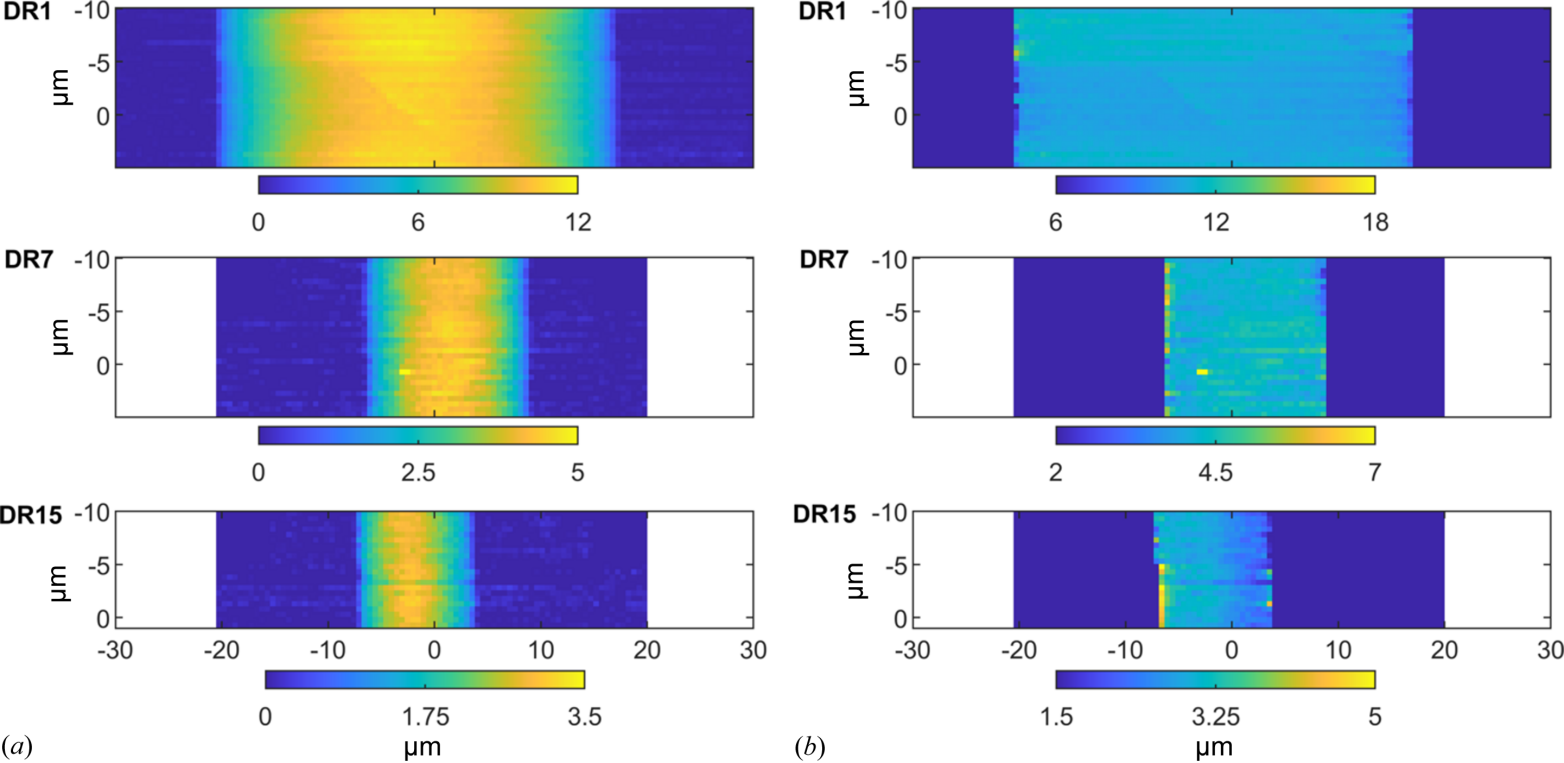
Scan maps (unit 10^5^ detector counts) after line-by-line background subtraction of the down-sampled datasets (in Fig. S2). (*b*) Scan maps (unit 10^5^ detector counts) after radial path-length normalization of the background data [in Fig. 2 ▸(*a*)]. The visualized range of values between the minimum and maximum are the same for each sample in (*a*) and (*b*).

In Fig. 3 ▸(*a*), we present the mapped total scattering intensity of the three different fibers after preprocessing and background subtraction (the analyzed area for DR15 includes only the vertical range −10 to 0 µm since it drifted out of the field of view, see Fig. S1). Corresponding path-length normalized intensity maps are presented in Fig. 3 ▸(*b*). The path-length normalized maps show an essentially constant intensity across all three fibers from which we can conclude that the fibers are homogeneous, without voids, on the 500 nm length scale. Below, all data presented have been path-length corrected.

**Figure 4:**
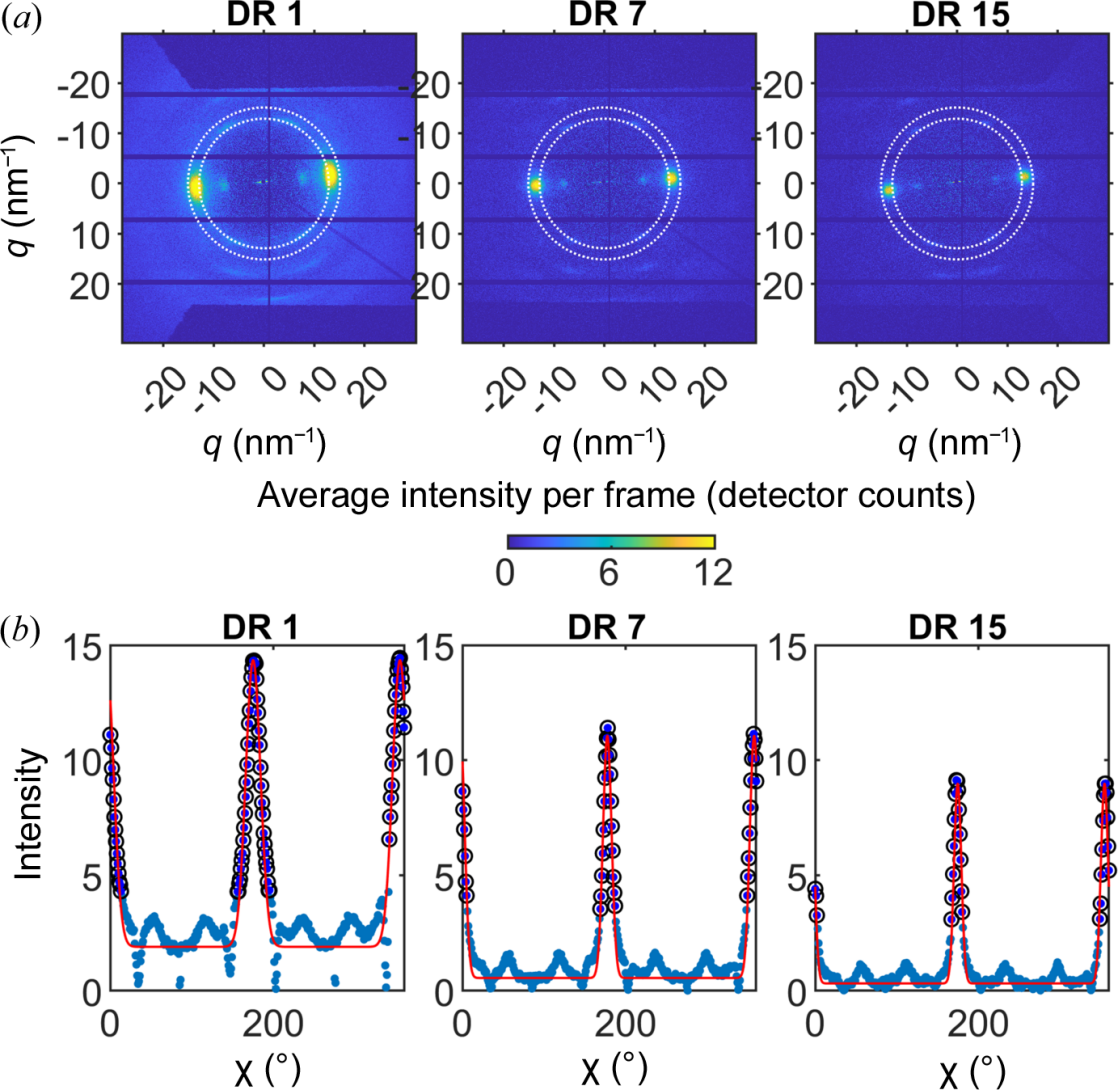
(*a*) Average frame diffraction pattern after background normalization and path-length normalization. Dotted white circles on the patterns indicate the *q*-band selected for azimuthal integration. (*b*) Gaussian function fitted to the average frame diffraction patterns. Blue points show the azimuth integrated data within the selected *q*-band. Black circles mark the data used to fit the Gaussian functions (red lines). Fixed and fitted parameters are reported in each subplot.

The average diffraction patters (averaged over all lines) for the three fibers after the radial path-length normalization step are shown in Fig. 4 ▸(*a*). The three fibers show very similar patterns, and they are diffraction patterns consistent with those obtained previously from the same fibers with a laboratory source instrument (Gubitosi *et al.*, 2021 ▸). The narrow diffraction spots imply a high degree of crystal orientation, with the cellulose chains oriented in the fiber direction. The strong equatorial peaks at *q* ≃ 14.5 nm^−1^ can be assigned to the (110) reflection, assuming the crystal structure of cellulose II (Langan *et al.*, 2001 ▸; French, 2014 ▸). With focus on this strong reflection, we have plotted the intensity within the narrow *q* band 13.2–15.6 nm^−1^ versus the azimuthal angle (θ) in Fig. 4 ▸(*b*). Aside from the main (110) peaks (at approximately 0 and 180°, respectively), this *q* band also includes four minor peaks that are neglected in the further analysis.

Apart from the average crystal orientation, the azimuthal plot also carries information on the width of the orientation distribution (*i.e.* the degree of crystal orientation) from the peak width. In our recent works, using our in-house laboratory-source instrument (Gubitosi *et al.*, 2021 ▸; Gentile *et al.*, 2022 ▸), we were able to separate scattering contributions coming from crystalline and amorphous domains within the fibers. Peaks could be fitted by a superposition of two Gaussian functions with significant difference in their widths (standard deviation). The narrow peak was identified as coming from the highly oriented crystalline domains while the broad component was identified with the more disordered amorphous domains. Access to the crystalline and amorphous scattering separately also allowed for an accurate evaluation of the average fiber crystallinity (Gentile *et al.* 2022 ▸), *i.e.* the volume fraction (*f*_c_) that, for the present fibers, were determined to ϕ_c_ = 0.4 for DR1 and ϕ_c_ = 0.6 for both DR7 and DR15.

In the present dataset, the SNR did not allow for a sufficiently accurate determination of the amorphous scattering contribution, as we have done in previous work (Gubitosi *et al.*, 2021 ▸; Gentile *et al.* 2022 ▸), and we therefore focus only on the crystalline contribution associating each peak with a single Gaussian function. Thus the function *I*(θ) used here was
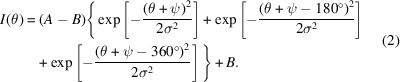
Here, *A* is the peak amplitude, σ is the standard deviation, ψ is an offset angle due to fiber tilting and *B* is a factor describing the background. The peak amplitude value *A* was calculated from the data in each frame and a mean value of the intensity between the main peaks (at angles 70–100°) was used as an estimate for the background amplitude *B*. Values of ψ and σ were extracted during the data-fitting procedure. Data points, included in the fit, are indicated by the black open circles in Fig. 4 ▸(*b*). Data were excluded for angles 28–152° and 200–342° for two main reasons: (1) for simplicity, the four minor peaks at approximately 60, 120, 240 and 300°, respectively, were excluded from the fit; (2) to restrict the fit to contributions coming only from the crystalline domains. The red solid lines in Fig. 4 ▸(*b*) represent the best fits of equation (2) to the data. Parameter values obtained are given in Table 1 ▸. As can be seen, *s* decreases with increasing DR, and the values are overall consistent with previous work (Gentile *et al.*, 2022 ▸). However, the σ values obtained here are systematically 10–20% smaller. We attribute such a difference to the fact that WAXS data here were measured on single fibers while the analysis of Gentile *et al.* (2022 ▸) was made on bundles of fibers, so that those σ values also included a small variation in the fiber orientation.

After confirmation that the average degree of crystal orientation, as quantified by a standard deviation σ of the orientation distribution, is consistent with previous work, we now turn to address possible variations among the fibers. When scanning across the fibers in the direction perpendicular to the axis, we probe different parts of the fiber [Fig. 2 ▸(*a*)]. In the *x* = 0 position, the beam probes the whole fiber thickness from *r* = 0 to *r* = *R*, where 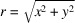 is the radial coordinate [Fig. 2 ▸(*a*)]. On the other hand, when the beam is close to the fiber edge (|*x*| ≲ *R*), only the near-surface region, with *r* ≃ *R*, is probed.

**Figure 5:**
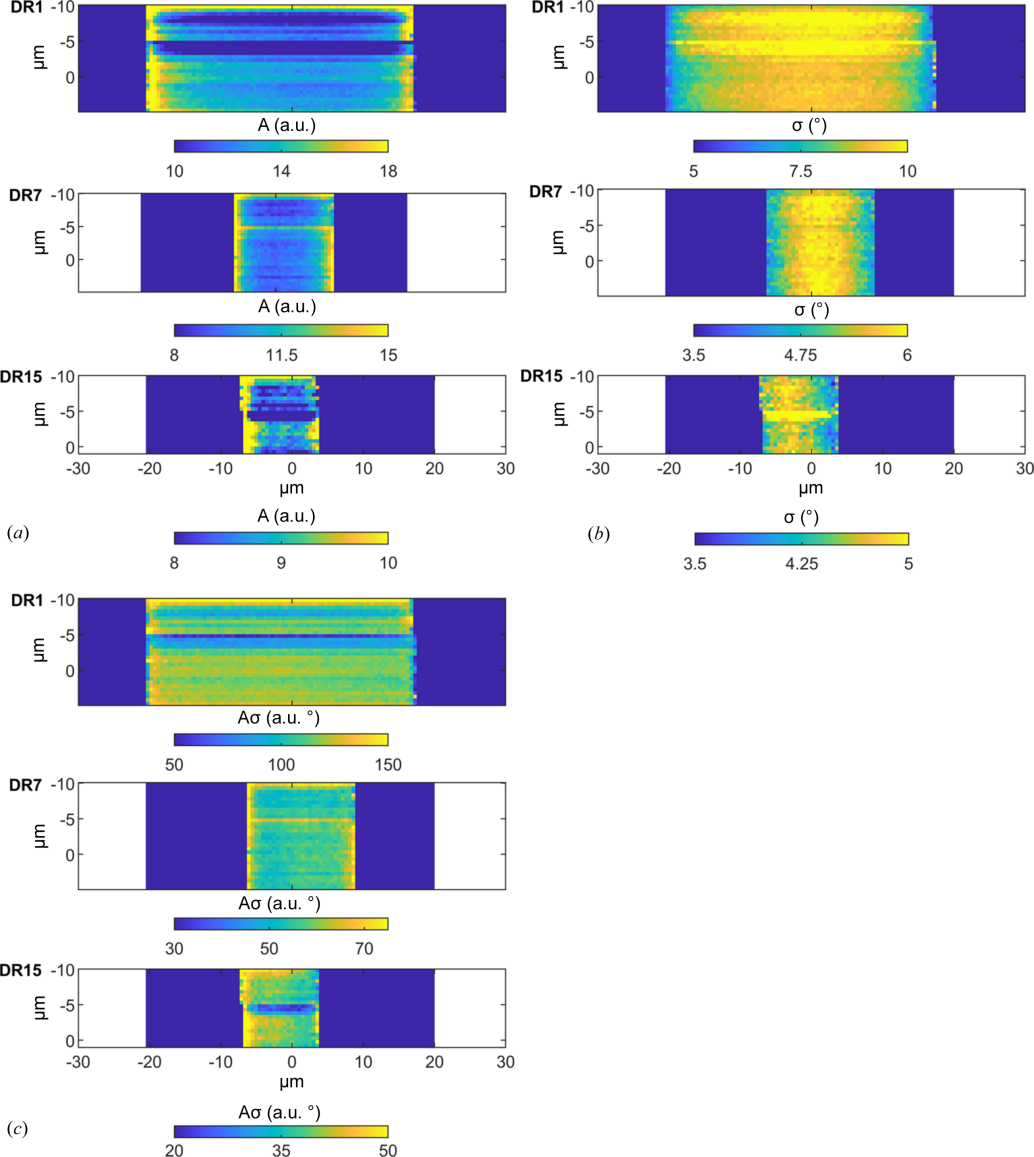
(*a*) Scan maps showing the variation of peak amplitude, *A*. (*b*) Scan maps showing the variation of standard deviation, σ. (*c*) Scan maps of the product *Aσ*, being a measure of the fiber crystallinity.

In Figs. 5 ▸(*a*) and 5 ▸(*b*), we show maps of the peak amplitude (*A*) and the standard deviation (σ) distributions, respectively. As can be seen, both parameters depend on *x*, but in the opposite way. *A* takes a minimum value at *x* = 0, while σ has a maximum. The latter implies that the variation in crystal orientation is broader in the center of the fibers compared with near the surface. This is clear for DR1 and DR7, while for DR15 the variation is minor, if at all.

The regenerated cellulose fibers are semi-crystalline materials with crystalline domains embedded in an amorphous matrix. The crystallinity (*i.e.* the volume fraction, *f*_c_) of the material that is crystalline can be obtained from integrating separately the scattering intensity from the crystalline domains 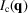 and normalize this with the total scattering, 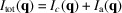, 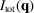 being the sum of contributions from the crystalline [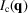] and amorphous [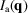] domains (de Jeu, 2016 ▸; Gentile *et al.*, 2022 ▸).
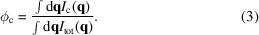
To a first approximation, the integral 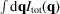 in the denominator is assumed to be a constant, not dependent on ϕ_c_ (de Jeu, 2016 ▸). This is confirmed by the constant total number of detector counts in each frame [Fig. 3 ▸(*b*)]. As the *q* range of the azimuthal analysis involves the most intense crystalline diffraction peak, we expect 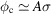 to hold to a good approximation. In Fig. 5 ▸(*c*), we present scan maps of the product *A*σ for the different fibers. As can be seen, *A*σ, and hence the crystallinity, are essentially homogeneous over the fibers. Therefore, the observed variation of *A* with *x* does not reflect any variation in crystallinity but is merely a consequence of the variation in σ.

**Figure 6:**
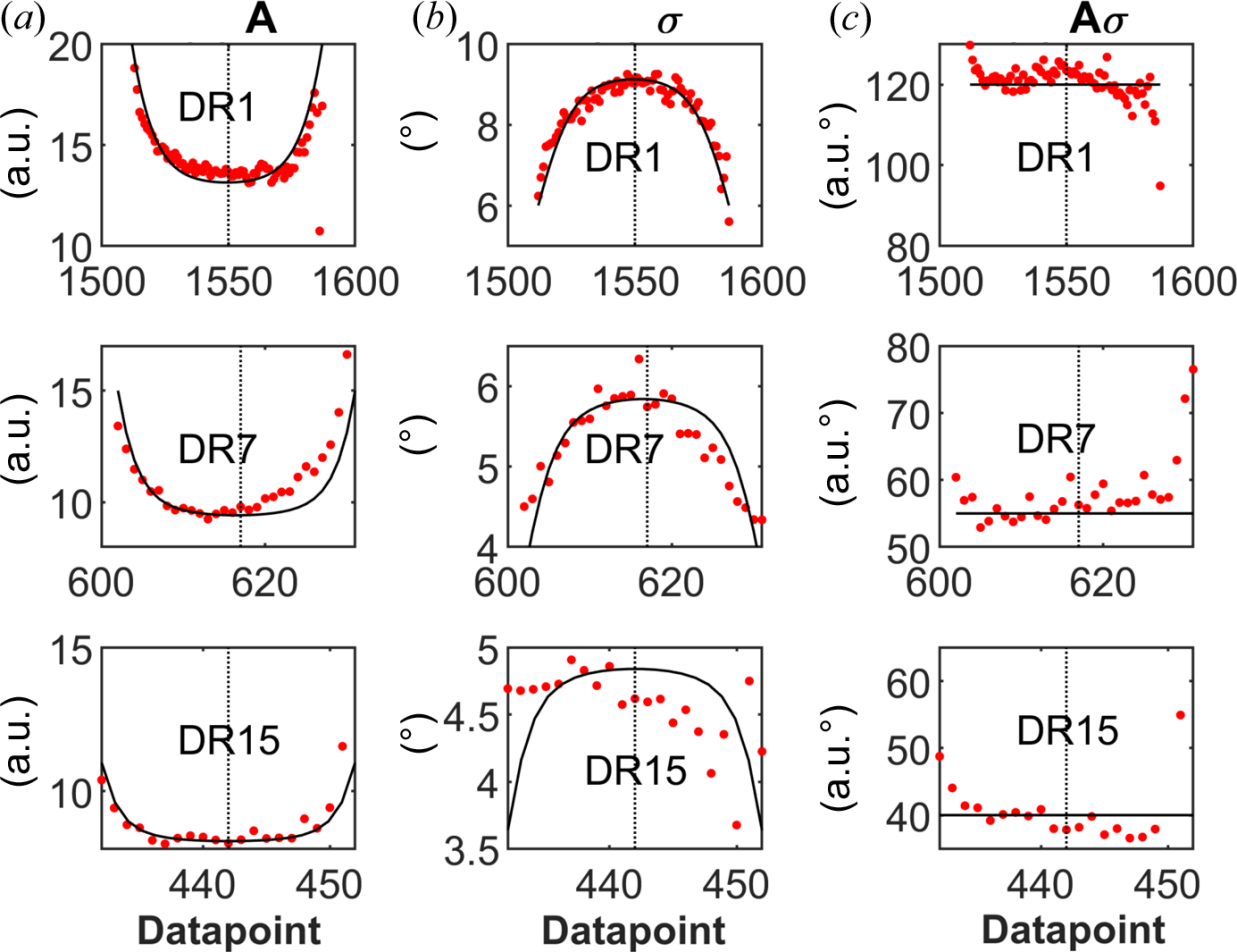
(*a*) Gaussian *A* variations along a single scan line in DR1, DR7 and DR15. The *A* values decrease radially from the fiber edges towards the fiber center (marked with dashed vertical line). (*b*) Gaussian σ variations along a single scan line in DR1, DR7 and DR15. The values increase radially from the fiber edges towards the fiber center (marked with dashed vertical line). (*c*) Product of *A* in (*a*) and σ in (*b*) as a measure of the total crystallinity, which is relatively constant along the scan lines.

For a more quantitative view, the variations in *A*, σ and *Aσ* across a single horizontal scan line in each fiber are plotted in Figs. 6 ▸(*a*)–6 ▸(*c*). For the DR1 and DR7 fibers, there are significant variations, approximately by 50%, in *A* and σ, while the product *Aσ* is essentially independent of *x*. On the other hand, for the DR15 fiber, the variations in *A* and σ are minor. Similar variations in *A* and σ are also observed for the other scan lines of the different fibers, as shown in Figs. S3–S5. In DR1 and DR7, the orientational order increases radially from the center of the fibers towards the edges. For DR15, the variation is minor.

**Figure 7:**
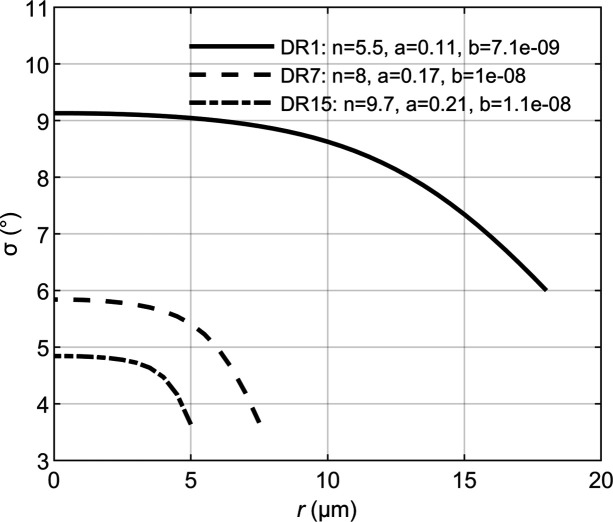
Radial profiles of the standard deviation σ(*r*) describing the degree of crystal orientation in the fibers. σ(*r*) has the functional form of equation (4), and the parameters for the different draw ratios are given in the figure, where *a* has the dimension 1/degrees and *b* has the dimension 1/(degrees µm^*n*^). *r* = 0 corresponds to the center of the fibers.

From the measured data in Figs. 6 ▸(*a*)–6 ▸(*c*), we have estimated how σ and *A* vary with *r*. We have assumed the following functional form
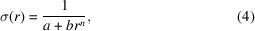
where *a* and *b* are constants. The corresponding function for the amplitude *A*(*r*) is then simply given by 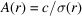, assuming *Aσ* to be constant, equal to *c*. To compare with the experimentally measured average 〈σ〉 and how it varies with the *x* coordinate [Fig. 5 ▸(*b*)], we performed the following numerical calculation. For a given value of *x*, we varied *y* from −*y*′ to *y*′ [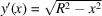, see Fig. 2 ▸(*c*)] in *N_y_* = 21 equally spaced points. For every value of *y_i_* (index *I* varying from 1 to *N_y_*), we calculate σ_*i*_ according to equation (4), where 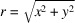. Finally, we obtained the average σ for the given *x* value from 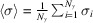. This calculation of the average σ was performed for varying *x* from *x* = −*R* to *x* = *R*, in steps of 0.5 µm, to obtain a numerical expression of σ(*x*). The parameters *a*, *b* and *n* were adjusted to obtain a σ versus *x* curve that resembles the experimental curve for the given draw ratio. Calculated curves, with parameters adjusted to agree with experimental data, are shown as solid lines in Fig. 6 ▸(*a*)–6 ▸(*c*). As can be seen, it is possible to obtain a reasonable description of the measured *A* and σ profiles, assuming the functional form σ(*r*) function of equation (4). The corresponding σ(*x*) profiles used in the calculations are presented in Fig. 7 ▸.

The observed radial gradients in crystal orientation can be qualitatively understood from considering the spinning process. Inside the spinneret, extrusion of the viscoelastic spindope introduces a velocity gradient which by symmetry is zero in the center and highest near the wall. Because of the high shear rate, chains in the vicinity of the wall align and stretch in the velocity direction. Similarly, drawing the viscoelastic fluid that exits the spinneret in the air gap results in a radial velocity profile with a maximum velocity in the center and a lower velocity on the surface. This velocity profile leads to thinning, but also to an increased alignment of chains near the surface due the higher shear rates. Interestingly, our data indicate that the crystallinity is homogeneous and does not vary with *r*. We note that the DR1 fibers also show a significant degree of crystal orientation, showing that considerable chain orientation occurs already in the extrusion step.

## 4. Conclusions

We have shown that it is possible to investigate structural variations of regenerated semi-crystalline cellulose fibers using an X-ray nano probe. The present experiments illustrate the challenges but also the possibilities in studying an organic material of small dimensions with an intense and focused X-ray beam. The present Ioncell-F textile fibers were found to be homogeneous in terms of crystallinity, but with a radial gradient in the crystal orientation here quantified as a standard variation σ(*r*) of an assumed Gaussian distribution. σ(*r*) were found to be maximum in the center of the fibers (*r* = 0) and monotonically decrease towards the fiber surface. We attribute this to the velocity gradients in the sample from the extrusion and from the drawing. This additional structural information, on the sub-micrometre length scale, may be of use in the design of optimal spinning conditions and in the theoretical modeling of the fiber mechanical properties.

## Supplementary Material

Supporting figures . DOI: 10.1107/S205225252400383X/ti5030sup1.pdf

## Figures and Tables

**Table 1:** Parameter values (peak amplitude *A*, standard deviation σ, offset angle ψ due to fiber tilting, background *B*) obtained from the Gaussian fit [equation (2)] to the data in Fig. 3 ▸(*b*) For the fitted parameters, the 95% confidence bounds are reported in parentheses.

	*A* (a.u.)†	σ (°)	ψ (°)	*B* (a.u.)†
DR1	12.4	9.0 (8.8, 9.3)	5.0 (5.2, 4.7)	1.9
DR7	10.5	5.3 (5.1, 5.5)	2.5 (2.7, 2.3)	0.55
DR15	8.7	4.5 (4.1, 4.8)	5.4 (5.8, 5.0)	0.31

† The arbitrary unit values of *A* and *B* are all on the same scale, respectively.

## References

[bb1] Asaadi, S., Hummel, M., Ahvenainen, P., Gubitosi, M., Olsson, U. & Sixta, H. (2018). *Carbohydr. Polym.* **181**, 893–901. 10.1016/j.carbpol.2017.11.062 29254051

[bb2] Bialik, E., Stenqvist, B., Fang, Y., Östlund, Å., Furó, I., Lindman, B., Lund, M. & Bernin, D. (2016). *J. Phys. Chem. Lett.* **7**, 5044–5048. 10.1021/acs.jpclett.6b02346 27973886

[bb3] Carbone, D., Kalbfleisch, S., Johansson, U., Björling, A., Kahnt, M., Sala, S., Stankevic, T., Rodriguez-Fernandez, A., Bring, B., Matej, Z., Bell, P., Erb, D., Hardion, V., Weninger, C., Al-Sallami, H., Lidon-Simon, J., Carlson, S., Jerrebo, A., Norsk Jensen, B., Bjermo, A., Åhnberg, K. & Roslund, L. (2022). *J. Synchrotron Rad.* **29**, 876–887. 10.1107/S1600577522001333 PMC9070697 35511021

[bb5] Davies, R. J. (2003). *J. Mater. Sci.* **38**, 2105–2115.

[bb4] Davies, R. J., Burghammer, M. & Riekel, C. (2006). *Macromolecules*, **39**, 4834–4840.

[bb6] French, A. D. (2014). *Cellulose*, **21**, 885–896. 10.1007/s10570-013-0051-z PMC3979627 24729665

[bb101] Gentile, L., Sixta, H., Giannini, C. & Olsson, U. (2022). *IUCrJ*, **9**, 492–496. 10.1107/S205225252200570X PMC9252157 35844479

[bb7] Gubitosi, M., Asaadi, S., Sixta, H. & Olsson, U. (2021). *Cellulose*, **28**, 2779–2789.

[bb8] Gubitosi, M., Nosrati, P., Koder Hamid, M., Kuczera, S., Behrens, M. A., Johansson, E. G. & Olsson, U. (2017). *R. Soc. Open Sci.* **4**, 170487–11. 10.1098/rsos.170487 PMC5579112 28878996

[bb9] Idström, A., Gentile, L., Gubitosi, M., Olsson, C., Stenqvist, B., Lund, M., Bergquist, K. E., Olsson, U., Köhnke, T. & Bialik, E. (2017). *Cellulose*, **24**, 3645–3657.

[bb10] Jeu, W. H. de (2016). *Basic X-ray Scattering for Soft Matter.* Oxford University Press.

[bb11] Johansson, U., Carbone, D., Kalbfleisch, S., Björling, A., Kahnt, M., Sala, S., Stankevic, T., Liebi, M., Rodriguez Fernandez, A., Bring, B., Paterson, D., Thånell, K., Bell, P., Erb, D., Weninger, C., Matej, Z., Roslund, L., Åhnberg, K., Norsk Jensen, B., Tarawneh, H., Mikkelsen, A. & Vogt, U. (2021). *J. Synchrotron Rad.* **28**, 1935–1947. 10.1107/S1600577521008213 PMC8570223 34738949

[bb12] Klemm, D., Heublein, B., Fink, H.-P. & Bohn, A. (2005). *Angew. Chem. Int. Ed.* **44**, 3358–3393. 10.1002/anie.200460587 15861454

[bb13] Langan, P., Nishiyama, Y. & Chanzy, H. (2001). *Biomacromolecules*, **2**, 410–416. 10.1021/bm005612q 11749200

[bb14] Marsh, J. T. & Wood, F. C. (1942). *An Introduction to the Chemistry of Cellulose.* London: Chapman & Hall.

[bb15] Martin-Bertelsen, B., Andersson, E., Köhnke, T., Hedlund, A., Stigsson, L. & Olsson, U. (2020). *Polymers*, **12**, 342. 10.3390/polym12020342 PMC7077394 32033419

[bb16] Riekel, C., Burghammer, M. & Davies, R. (2010). *IOP Conf. Ser. Mater. Sci. Eng.* **14**, 012013.

[bb17] Riekel, C. & Davies, R. J. (2005). *Curr. Opin. Colloid Interface Sci.* **9**, 396–403.

[bb18] Röder, T., Moosbauer, J., Kliba, G., Schlader, S., Zuckerstätter, G. & Sixta, H. (2009). *Lenzing. Ber.* **87**, 98–105.

[bb19] Rosenau, T., Potthast, A., Sixta, H. & Kosma, P. (2001). *Prog. Polym. Sci.* **26**, 1763–1837.

[bb20] Roth, S., Burghammer, M., Janotta, A. & Riekel, C. (2003). *Macromolecules*, **36**, 1585–1593.

[bb21] Siliqi, D., De Caro, L., Ladisa, M., Scattarella, F., Mazzone, A., Altamura, D., Sibillano, T. & Giannini, C. (2016). *J. Appl. Cryst.* **49**, 1107–1114. 10.1107/S1600576716010396 PMC4970496 27504077

[bb22] Sixta, H., Michud, A., Hauru, L., Asaadi, S., Ma, Y., King, A. W. T., Kilpeläinen, I. & Hummel, M. (2015). *Nord. Pulp Pap. Res. J.* **30**, 43–57.

[bb23] Swatloski, R. P., Spear, S. K., Holbrey, J. D. & Rogers, R. D. (2002). *J. Am. Chem. Soc.* **124**, 4974–4975. 10.1021/ja025790m 11982358

[bb24] Tu, H., Zhu, M., Duan, B. & Zhang, L. (2021). *Adv. Mater.* **33**, 2000682. 10.1002/adma.202000682 32686231

[bb25] Wang, S., Lu, A. & Zhang, L. (2016). *Prog. Polym. Sci.* **53**, 169–206.

[bb26] Woodings, C. (2001). *Regenerated Cellulose Fibres*, 1st ed. pp. 1–21. Boston: Woodhead Publishing Limited.

