# Supplementary material for: Scanning WAXS microscopy of regenerated cellulose fibers at mesoscopic resolution
Source: IUCrJ. 2024 Jun 11;11(Pt 4):570–7. doi: 10.1107/S205225252400383X (PMC11220875; doi:10.1107/S205225252400383X)
Supplement: Supplementary file 1 [file m-11-00570-sup1.pdf]

IUCrJ

Volume 11 (2024)

Supporting information for article:

Scanning WAXS Microscopy of Regenerated Cellulose Fibers at Mesoscopic Resolution

Sara Johansson, Francesco Scattarella, Sebastian Kalbfleisch, Ulf Johansson, Christopher Ward, Crispin Hetherington, Herbert Sixta, Stephen Hall, Cinzia Giannini and Ulf Olsson

In this supporting information we present raw scan maps before post-processing (Figure S1) and scan maps after the I_0 normalization and sample tilt correction (Figure S2). Finally, in Figure S3 we present line-by-line variations of the amplitude (A) and standard deviation (σ), respectively, of the peaks in a azimuthal plot, and the product $A\sigma$, for the DR1 fiber.

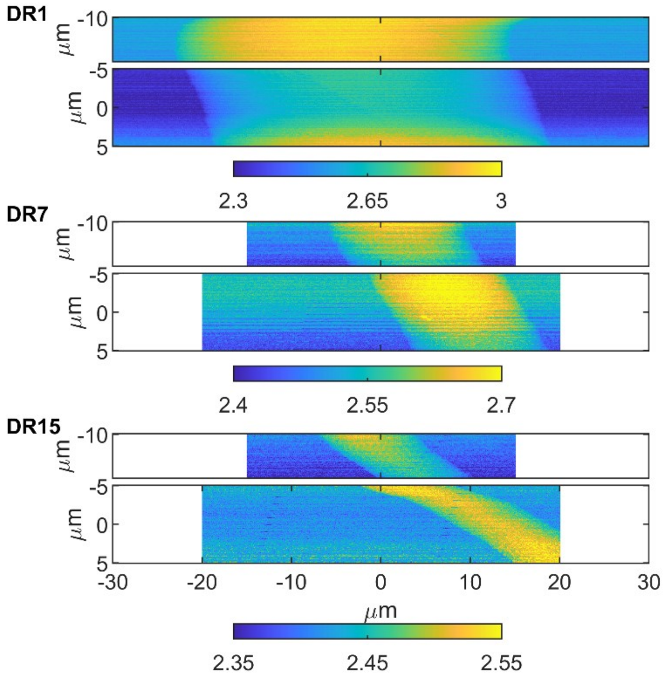

Figure S1. Scanning WAXS maps showing the total scattered intensity (unit 10^5 detector counts) for each measured frame. Two data sets were acquired for each fiber.

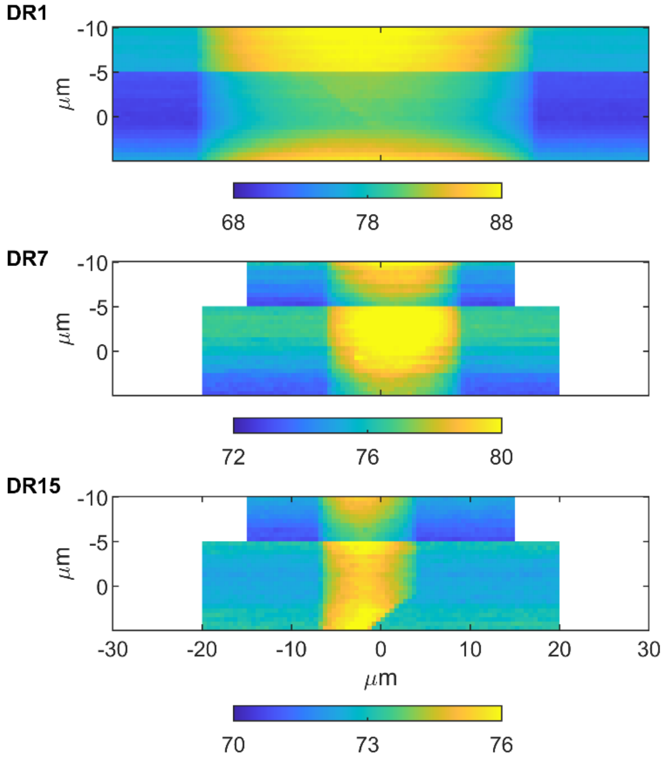

Figure S2. Scan maps (unit 10^5 detector counts) of down sampled datasets (sum of 5x5 frames in each pixel) after the I_0 normalization and sample tilt correction of the data in Figure S1. The tilt correction was performed by measuring the tilt of the fiber, whereafter appropriate numbers of pixels were shifted from the beginning to the end of each scan line.

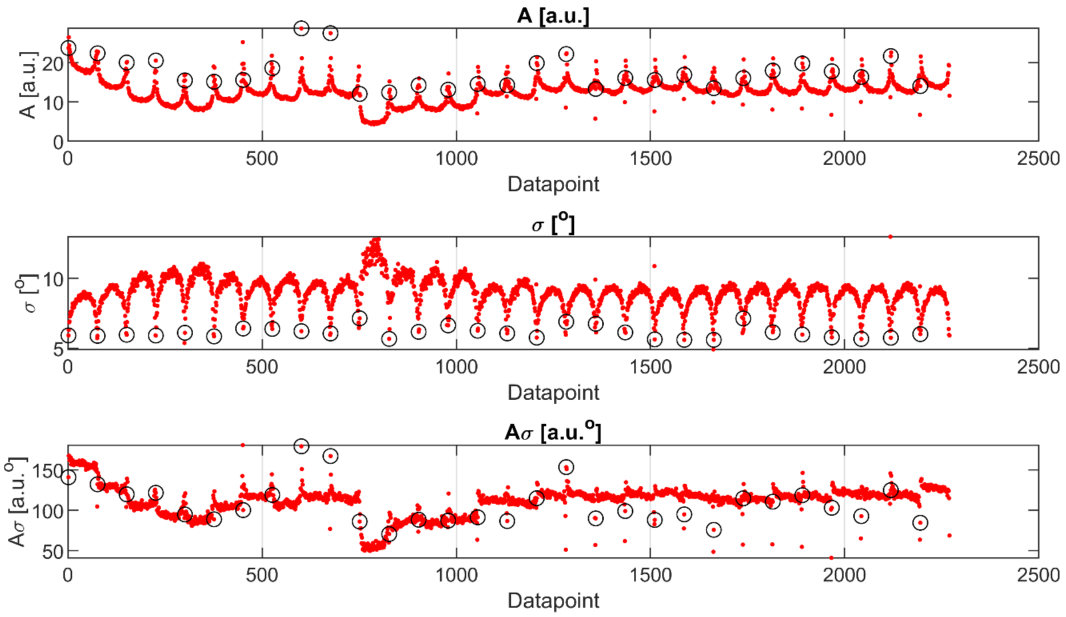

Figure S3. Line-by-line variation of amplitude A, standard deviation σ and the product of these two values in the DR1 fiber. Black circles mark the beginning of a new line.

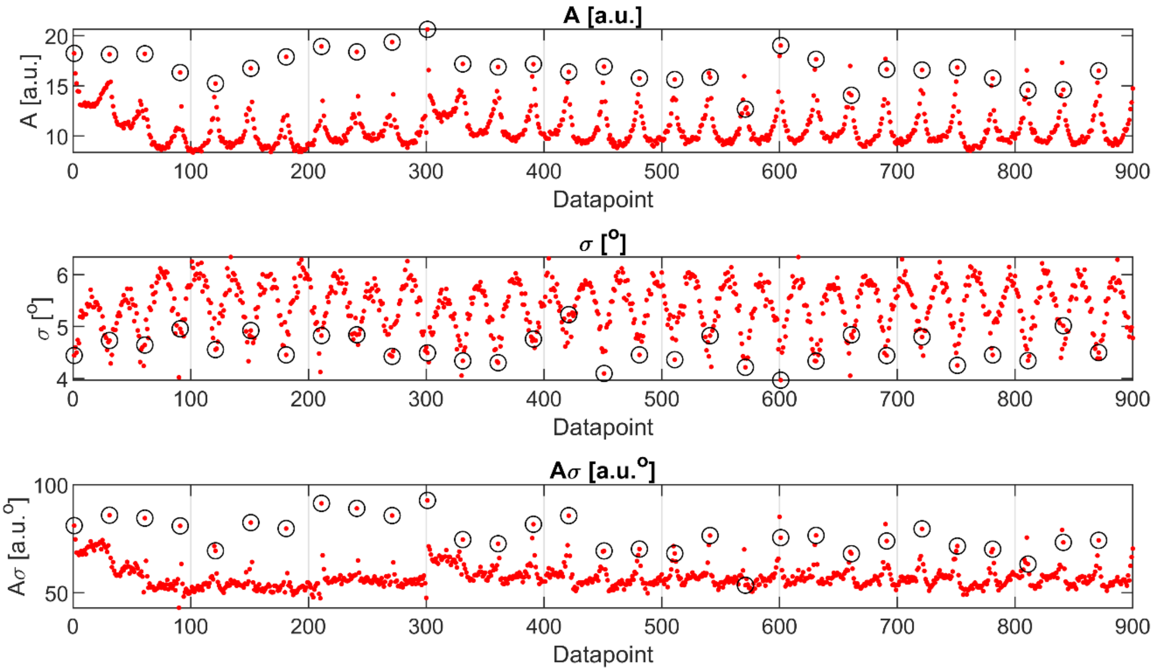

Figure S4. Line-by-line variation of amplitude A, standard deviation σ and the product of these two values in the DR7 fiber. Black circles mark the beginning of a new line.

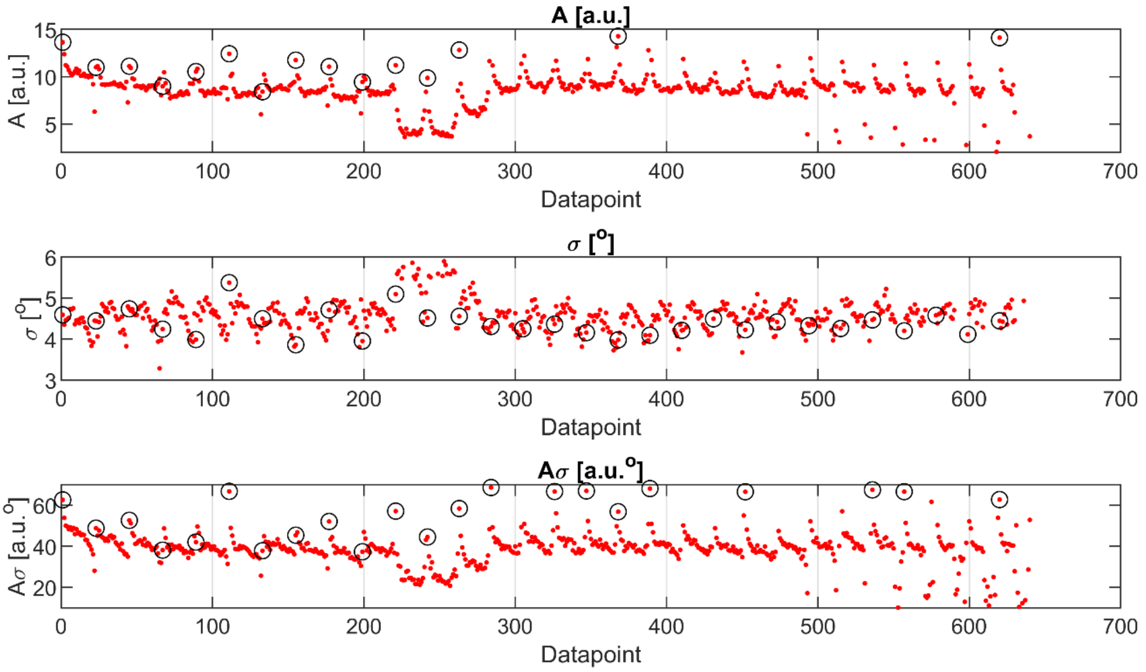

Figure S5 Line-by-line variation of amplitude A, standard deviation σ and the product of these two values in the DR15 fiber. Black circles mark the beginning of a new line.
